# Renal Denervation Reduced Ventricular Arrhythmia After Myocardial Infarction by Inhibiting Sympathetic Activity and Remodeling

**DOI:** 10.1161/JAHA.118.009938

**Published:** 2018-10-13

**Authors:** Wen‐hui Zhang, Qi‐na Zhou, Yan‐mei Lu, Yao‐dong Li, Ling Zhang, Jiang‐hua Zhang, Qiang Xing, Wen‐kui Lv, Xin‐chun Cheng, Ge‐ge Zhang, Xue‐sheng Wang, Qi Gu, Xue Lou, Buajier Guli, Bao‐peng Tang, Xian‐hui Zhou

**Affiliations:** ^1^ Cardiac Pacing and Electrophysiological Division The First Affiliated Hospital of Xinjiang Medical University Urumqi Xinjiang China; ^2^ Xinjiang Key Laboratory of Medical Animal Model Research Clinical Medical Research Institute The First Affiliated Hospital of Xinjiang Medical University Urumqi Xinjiang China; ^3^ Geriatric Center The People's Hospital of Xinjiang Uygur Autonomous Region Urumqi Xinjiang China

**Keywords:** myocardial infarction, remodeling, renal nerves, sympathetic nerve activity, Arrhythmias, Electrophysiology, Sudden Cardiac Death, Ventricular Fibrillation

## Abstract

**Background:**

Ventricular arrhythmia after myocardial infarction is the most important risk factor for sudden cardiac death, which poses a serious threat to human health. As the correlation between autonomic nervous systemic dysfunction and heart rhythm abnormality has been gradually revealed, remedies targeting autonomic nervous system dysfunction, especially the sympathetic nerve, have emerged. Among them, renal denervation is noted for its powerful effect on the inhibition of sympathetic nerve activity. We aim to investigate whether renal denervation can reduce ventricular arrhythmia after myocardial infarction and thus decrease the risk of sudden cardiac death. In addition, we explore the potential mechanism with respect to nerve activity and remodeling.

**Methods and Results:**

Twenty‐four beagles were randomized into the control (n=4), renal denervation (n=10), and sham (n=10) groups. Permanent left anterior descending artery ligation was performed to establish myocardial infarction in the latter 2 groups. Animals in the renal denervation group underwent both surgical and chemical renal denervation. Compared with dogs in the sham group, dogs in the renal denervation group demonstrated attenuated effective refractory period shortening and inhomogeneity, flattened restitution curve, increased ventricular threshold, and decreased ventricular arrhythmia. Heart rate variability assessment, catecholamine measurement, and nerve discharge recordings all indicated that renal denervation could reduce whole‐body and local tissue sympathetic tone. Tissue analysis revealed a significant decrease in neural remodeling in both the heart and stellate ganglion.

**Conclusions:**

Surgical and chemical renal denervation decreased whole‐body and local tissue sympathetic activity and reversed neural remodeling in the heart and stellate ganglion. Consequently, renal denervation led to beneficial remodeling of the electrophysiological characteristics in the infarction border zone, translating to a decrease in ventricular arrhythmia after myocardial infarction.

**Clinical Perspective**

**What Is New?**

- According to our study, for myocardial infarction patients with high risk of ventricular arrhythmia after percutaneous coronary intervention, renal denervation should be considered to reduce that potential risk.

**What Are the Clinical Implications?**

- Renal denervation may decrease the incidence of sudden cardiac death after myocardial infarction and thus reduce the disease and economic burden associated with myocardial infarction.

## Introduction

Sudden cardiac death (SCD) is a leading cause of mortality worldwide, and despite improvements in resuscitation, SCD significantly affects human health.^1^ Coronary heart disease, followed by cardiomyopathies, inherited arrhythmia syndromes, and valvular heart disease, are known as the most common pathology underlying SCD.^2^ Epidemiological studies have suggested that such rapid deaths are caused by lethal ventricular arrhythmias (VAs) in the setting of underlying coronary heart disease mostly as a result of ventricular tachyarrhythmia after previous myocardial infarction (MI).^3^ Both sympathetic nerve (SN) overactivation^4^, ^5^ and regional cardiac hyperinnervation^6^ trigger VA post‐MI (VAPMI). Reduced β‐receptor blocker (B‐blocker) was associated with increased susceptibility to ventricular fibrillation (VF) in a canine model of healed MI.^7^ A clinical study also demonstrated that B‐blocker reduced mortality by 40% in patients with MI.^8^ Β‐blocker exerts this cardioprotective effects by targeting the nervous system and the end effectors of the heart. Understandably, regimens targeting the sympathetic nervous system have become an area of research interest and gained promising results.^9^, ^10^

Renal denervation (RDN) decreases renal norepinephrine spillover. RDN simultaneously reduces whole‐body norepinephrine spillover by 42% and muscle SN activity, which highlights the possibility that inhibition of afferent renal nerve activity may contribute to a reduction in the central sympathetic drive.^11^ Based on these findings, subsequent clinical studies have used RDN in refractory hypertension and produced inconsistent results.^12^, ^13^, ^14^ Consequently, the efficacy of RDN has remained questionable. A recent multicenter study, however, provided powerful proof for the blood pressure–lowering efficacy of RDN.^15^

As mentioned, VAPMI is driven by sympathetic responses. We hypothesized (1) that RDN can significantly attenuate sympathetic tone, with a resulting decrease in VAPMI, and (2) that nerve sprouting in the infarction border zone (IBZ) can increase the probability of VAPMI. The IBZ has been shown to have unique structural and electrophysiological remodeling attributes that contribute to the development of VAPMI. Consequently, we hypothesized that RDN can inhibit excessive nerve regeneration in the IBZ and was sufficient to decrease VAPMI. To test our hypotheses, we performed surgical and chemical RDN in canine models of MI. Because some studies have suggested that incomplete RDN might diminish the efficacy of RDN, we measured renal SN activity by stimulating the aorticorenal ganglia (ARG) and monitoring blood pressure to ensure the success of RDN. We also assessed sympathetic tone by directly recording neural discharge in addition to using other available approaches.

## Methods

The authors declare that all supporting data are available within the article and its online supplementary files.

### Ethics Statement

This study was performed in accordance with the National Institutes of Health *Guide for the Care and Use of Laboratory Animals*. The study protocol was approved by the Institutional Animal Care and Use Committee of the First Affiliated Hospital of Xinjiang Medical University (permit no. IACUC‐20150210‐13). All efforts were made to minimize animal suffering.

### Animal Model

Twenty‐four beagles were randomized into 3 groups. The RDN group (n=10) underwent ligation of the left anterior descending branch. One hour later, surgical and chemical RDN was performed. In brief, both kidneys were approached through a midabdominal incision. Bilateral kidneys were surgically denervated by cutting all visible nerves in the area of the renal hilum (surgical RDN), and then the area was moistened with a 20% phenol solution for 8 to 10 minutes (chemical RDN).^16^ The sham group (n=10) also underwent left anterior descending branch ligation and bilateral renal hilar exposure but with no further operation. The control group (n=4) underwent neither coronary ligation nor RDN.

### Confirmation of Intraoperative Efficacy of RDN

Arterial blood pressure was monitored via the left femoral artery sheath connected to a pressure transducer. While the renal artery was exposed, the tissue surrounding the junction of the renal artery and the aorta was bluntly and carefully dissected to locate the ARG.^17^ The ARG innervates the renal artery, and electrical stimulation of the ARG significantly increases arterial blood pressure.^18^ Before and 10 minutes after RDN, electrical stimulation delivered from a Grass stimulator (Astro‐Med Grass Technologies) at 20‐Hz frequency, 5‐ms pulse duration, and 12 V output was applied to the ARG.^17^ If the pressure‐increasing effect was weakened (systolic blood pressure increase ≤15 mm Hg), successful RDN was considered to be achieved.

### Open‐Chest Electrophysiological Examination

After the ligation location was decided, 1 quadripolar catheter and 1 decapolar catheter were sutured in parallel into the epicardium. For these 2 catheters, the midpoint of the electrodes was sutured to the presupposed ligation location. The catheters can separately record electrical activity of the infarction zone, the IBZ, and the noninfarction zone. In addition, a quadripolar catheter was used for monophasic action potential duration (MAPD) recording, and a decapolar catheter was used for measuring effective refractory periods (ERPs), VF inducibility, and VF threshold. The first 2 groups of animals underwent epicardial electrophysiological examination before and 1 hour and 4 weeks after coronary ligation.

### Dynamic ECG

A 24‐hour dynamic ECG (DEC) was performed in all animals at baseline. Next, the first 2 groups of dogs underwent DEC on the operation day after model establishment and RDN (RDN group). Thereafter, weekly 24‐hour DEC was performed for an additional 4 weeks. All DECs were recorded in ambulatory dogs to detect spontaneous VA. Ventricular tachycardia (VT) was defined by 3 consecutive extra ventricular beats with heart rate >120 beats/min and quantified as the number of episodes. The most important DEC data included VA and heart rate variability index.

### Nerve Discharge Recording

At 4 weeks after MI, the discharge of the cervical vagus nerve (VN) and inferior cardiac SN (emanating from the stellate ganglion [SG]) together with cardiac electrical activity were continuously recorded with PowerLab (Bio Amp; ADInstruments). The details of nerve separation and discharge recording methods have been reported previously by our team.^19^ All signals were analyzed with the Analysis Module of Lab Chart 8.0/proV7 software (Bio Amp; ADInstruments). To quantitatively assess nerve activity, we calculated 2 nerve discharge measurement indexes: root mean square (RMS), which is related to both nerve discharge amplitude and nerve discharge duration, and the proportion of nerve discharge duration of the total recording time course (Dt/Tt).

### Catecholamine Measurement

Blood samples of all dogs were collected before and 4 weeks after operation. Specimens of bilateral kidneys and IBZs of hearts were excised for catecholamine determination. Because the catecholamine content of biological specimens is small and catecholamine is easily degraded, the samples were processed and used for detection as soon as possible. To precisely assay epinephrine and norepinephrine levels, we used the ultraperformance liquid chromatography–tandem mass spectrometry.

### Tissue Analysis

After the completion of the final electrophysiological study, the heart, bilateral kidneys, and SGs were removed. The kidneys were harvested for further hematoxylin and eosin staining to observe injury to the renal artery and nerve. The ventricular tissues were cut centering the IBZ and with epicardial and endocardial aspects of the myocardium in each slice. The explanted heart tissues and bilateral SGs were subjected to western blotting and immunofluorescence assay or immunohistochemistry.

### Statistical Analysis

Measurement data that followed a normal distribution were expressed as mean±SD. Other data were reported as median (interquartile range [IQR]). In normally distributed data, repeated measures ANOVA was performed to compare data over time for each dependent variable measured at 3 time points: baseline, acute MI (AMI: 1 hour or the first 24 hours after MI) and 4 weeks after MI. Post hoc multiple comparisons were performed with the least significant difference method. In nonnormally distributed data, the Mann–Whitney rank test was applied to evaluate the differences. VF inducibility was compared with the Fisher exact probability test. *P*<0.05 was considered statistically significant. Statistical analysis were performed with SPSS Statistics v17 software (IBM Corp).

## Results

Three dogs in the RDN group and 2 dogs in the sham group died of VF after coronary ligation, and the data for the rest of the surviving animals were analyzed. All animals in the RDN group underwent successful surgical and chemical RDN. The blood pressure data are presented in Figure S1.

### Efficacy of RDN on Ventricular Repolarization

#### Effective Refractory Periods

**Figure 1:**
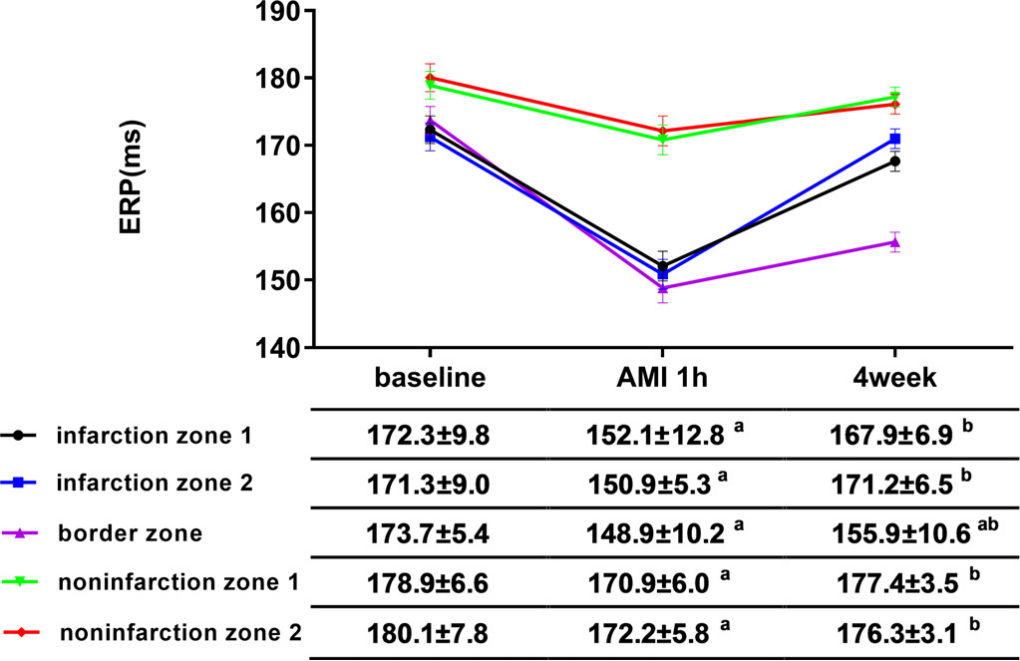
ERP changes across different locations. ERPs at baseline and 1 hour (AMI) and 4 weeks after MI are shown. ^a^ *P*<0.05 compared with baseline, ^b^ *P*<0.05 compared with AMI. AMI indicates acute myocardial infarction; ERP, effective refractory period.

ERPs slightly decreased from base to apex. In addition, compared with the periods at baseline, the periods significantly shortened during AMI (1 hour after ligation of the coronary artery), and this tendency was especially noted in infarction zones and the IBZ. By 4 weeks after MI, ERPs were restored to varying degrees, whereas the ERPs at the IBZ were still greatly reduced compared with those at baseline (Figure 1).

**Figure 2:**
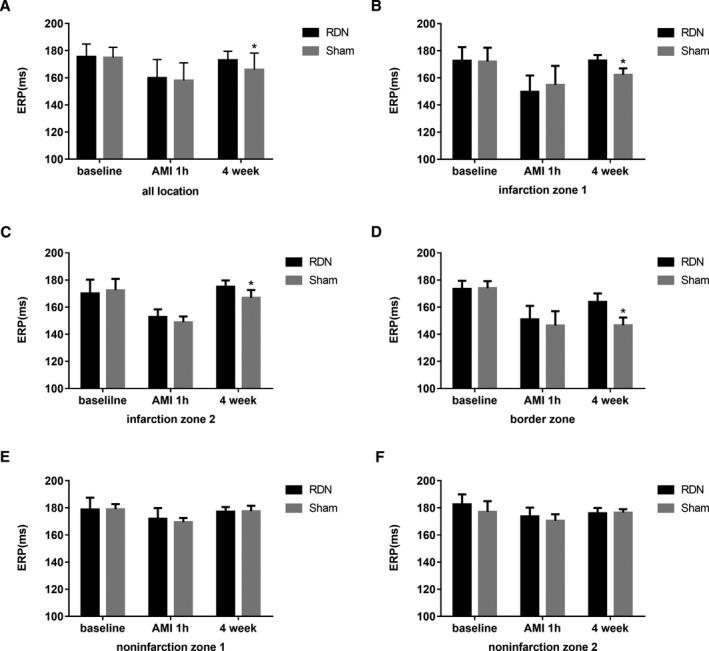
ERP differences between the RDN and sham groups at baseline and 1 hour (AMI) and 4 weeks after myocardial infarction. Both average ERP differences and ERP differences at each location are demonstrated. A, Average ERP differences at all locations; (B) ERP differences at infarction zone 1; (C) ERP differences at infarction zone 2; (D) ERP differences at the infarction border zone; (E) ERP differences at noninfarction zone 1; (F) ERP differences at noninfarction zone 2. *P<0.05 compared with the RDN group. AMI indicates acute myocardial infarction; ERP, effective refractory period; RDN, renal denervation.

**Figure 3:**
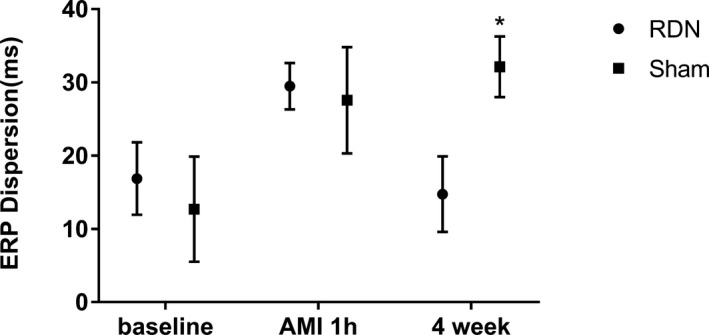
ERP dispersion differences between the RDN and sham groups at baseline and 1 hour (AMI) and 4 weeks after myocardial infarction. *P<0.05 compared with the RDN group. AMI indicates acute myocardial infarction; ERP, effective refractory period; RDN, renal denervation.

ERPs were similar in the RDN and sham groups at baseline. The same tendency was noted during AMI with no difference between the 2 groups. Four weeks later, ERPs were longer in the RDN group than in the sham group (173±6.5 versus 166±12.22; *P*<0.0001; Figure 2A). Regarding the specific location, infarction zone 1, infarction zone 2, and the IBZ demonstrated the same differences as described, and the difference was especially evident in the IBZ (Figure 2B–2D). No difference was seen in non–infarction zones (Figure 2E and 2F) either at baseline or during AMI. The animals in the RDN group had smaller ERP dispersion than animals in the sham group at 4 weeks after MI (median: 14 [IQR: 11.5–18] versus 31 [IQR: 29.5–33.5]; *P*<0.0001; Figure 3).

#### MAPD restitution characteristics

**Figure 4:**
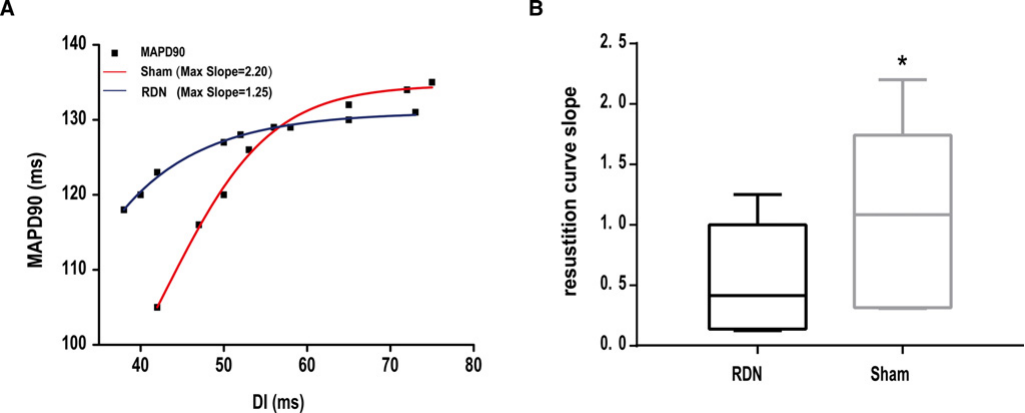
Comparison of the MAPD_90_ restitution curve and restitution curve slope between the RDN and sham groups 4 weeks after myocardial infarction. A, Difference in the MAPD_90_ restitution curves (A) and MAPD_90_ restitution curve slopes (B) of the RDN and sham groups. *P<0.05 compared with the RDN group. MAPD_90_ indicates 90% monophasic action potential duration; RDN, renal denervation.

The MAPD_90_ restitution curve at 4 weeks after MI from each group is shown in Figure 4A. The MAPD restitution curve was steeper in the sham group than in the RDN group. Accordingly, the slope of the restitution curve in the sham group was larger than that in the RDN group (median: 1.1 [IQR: 0.33–1.69] versus 0.42 [IQR: 0.15–1]; *P*=0.0352; Figure 4B).

### Effects of RDN on VA, VF Inducibility, and VF Threshold

#### Spontaneous VA

**Figure 5:**
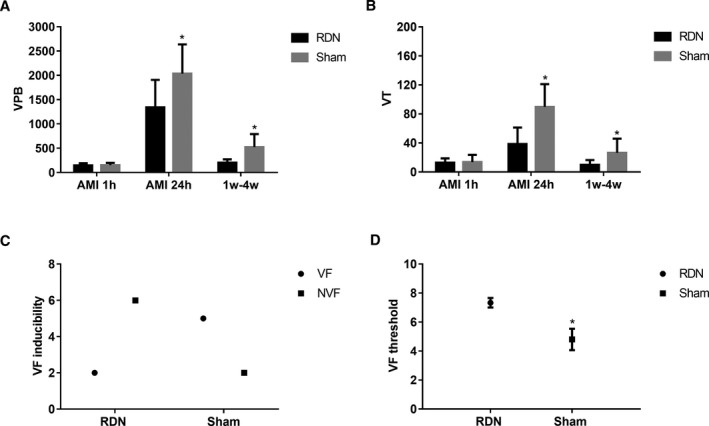
VPB and VT of the RDN and sham groups at AMI 1 hour, AMI 24 hours, and 1 to 4 weeks after MI and VF inducibility and VF threshold 4 weeks after MI. A, VPB differences of the RDN and sham groups. B, VT differences of the RDN and sham groups, C, Numbers of VF‐induced animals in the RDN and sham groups. D, VF threshold differences of the RDN and sham groups. *P<0.05 compared with the RDN group. AMI indicates acute myocardial infarction; MI, myocardial infarction; NS, no statistical significance; RDN, renal denervation; VF, ventricular fibrillation; VPB, ventricular premature beat; VT, ventricular tachycardia.

Ventricular premature beat (VPB) and VT were comparable in the RDN and sham groups 1 hour after coronary artery ligation. Following DEC, prompted VPB was markedly lower in the RDN group than in the sham group (1341.38±565.24 versus 2036.71±600.82; *P*=0.0380; Figure 5A) during AMI (first 24 hours after coronary ligation). Weekly 24‐hour DEC recording indicated that VPB in the RDN group at 4 weeks was markedly lower than that in the sham group (203±66.92 versus 524.71±265.96; *P*=0.03953; Figure 5A). Similarly, the difference in VT existed between the 2 groups both during the first 24 hours (38.62±22.62 versus 89.71±31.37; *P*=0.0415; Figure 5B) and during the following 4 weeks (9.88±6.56 versus 26.43±19.52; *P*=0.03613; Figure 5B) after AMI.

#### VF inducibility and VF threshold

VF was induced in 2 of 8 dogs in the RDN group and in 5 of 7 dogs in the sham group, albeit without statistical significance (Figure 5C). The VF threshold was significantly higher in animals in the RDN group than in animals in the sham group (median: 7 [IQR: 7–7.5] versus 4 [IQR: 4–6]; *P*=0.0437; Figure 5D).

### Effects of RDN on Sympathetic Activity

#### Systemic and local tissue catecholamine levels

**Figure 6:**
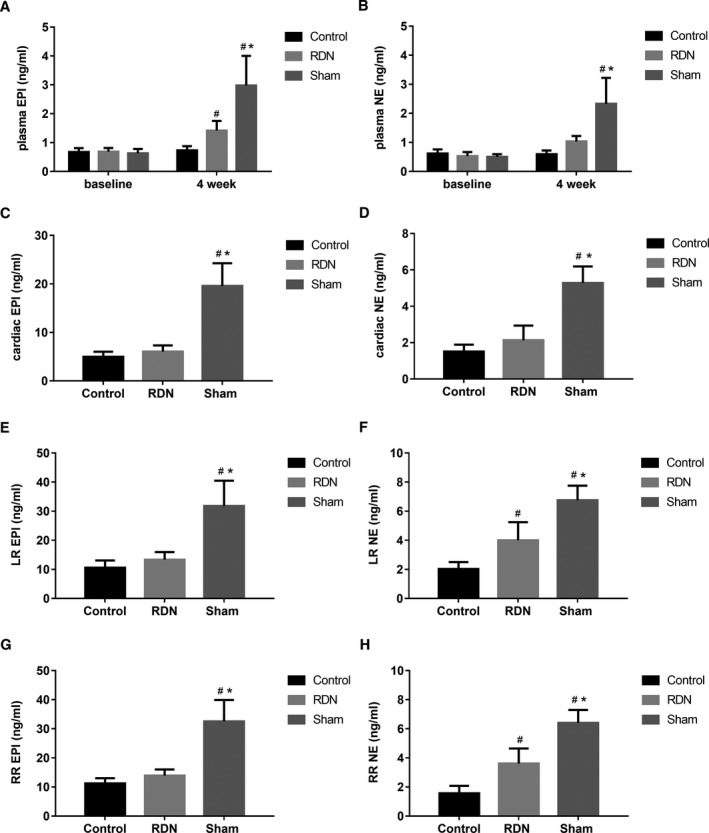
Plasma, cardiac, and bilateral renal catecholamine levels in the control, RDN, and sham groups at baseline and 4 weeks after myocardial infarction. Differences for the control, RDN, and sham groups for (A) plasma EPI, (B) plasma NE, (C) cardiac EPI, (D) cardiac NE, (E) LR EPI, (F) LR NE, (G) RR EPI, and (H) RR NE. ^#^ *P*<0.05 compared with the control group, *P<0.05 compared with the RDN group. EPI indicates epinephrine; LR, left renal; NE, norepinephrine; RDN, renal denervation; RR, right renal.

The plasma, cardiac, and bilateral renal levels of epinephrine and norepinephrine were measured. No obvious difference in baseline plasma catecholamine was observed among the control, RDN, and sham groups. The plasma catecholamine levels in the sham group were higher than those in the RDN and control groups 4 weeks after MI. In addition, the plasma catecholamine levels were significantly different between the RDN and control groups (epinephrine: RDN, 1.42±0.33 ng/mL; sham, 2.98±1.02 ng/mL; control, 0.74±0.14 ng/mL; *P*=0.0016; norepinephrine: RDN, 1.03±0.19 ng/mL; sham, 2.33±0.89 ng/mL; control, 0.59±0.13 ng/mL; *P*=0.0004; Figure 6A and 6B). Cardiac catecholamine levels in the control and RDN groups were evidently lower than those in the sham group (epinephrine: RDN, 6.03±1.27 ng/mL; sham, 19.56±4.71 ng/mL; control, 4.99±1.02 ng/mL; *P*<0.0001; norepinephrine: RDN, 2.13±0.81 ng/mL; sham, 5.29±0.91 ng/mL; control, 1.51±0.38; *P*<0.0001; Figure 6C and 6D). Bilateral renal epinephrine levels displayed the same difference as cardiac epinephrine levels (epinephrine, left renal: RDN, 13.35±2.58 ng/mL; sham, 31.76±8.73 ng/mL; control, 10.58±2.48 ng/mL; *P*<0.0001; right renal: RDN, 13.89±2.13 ng/mL; sham, 32.58±7.30 ng/mL; control, 11.25±1.76 ng/mL; *P*<0.0001; Figure 6E and 6G). However, bilateral renal norepinephrine levels in both the RDN and sham groups were higher than those in the control group, and RDN alleviated the increase (norepinephrine, left renal: RDN, 4.00±1.24 ng/mL; sham, 6.75±1.00 ng/mL; control, 2.03±0.48 ng/mL; *P*<0.0001; right renal: RDN, 3.61±1.03 ng/mL; sham, 6.41±0.88 ng/mL; control, 1.56±0.52 ng/mL; *P*<0.0001; Figure 6F and 6H).

#### Variability in heart rate

**Figure 7:**
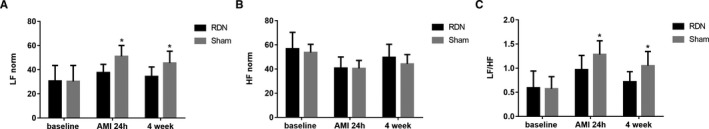
Frequency domain indexes of heart rate variability: LF norm, HF norm, and LF/HF in the RDN and sham groups at baseline and AMI 24 hours and 4 weeks after myocardial infarction. Differences for RDN and sham groups: (A) LF norm, (B) HF norm, and (C) LF/HF. *P<0.05 compared with the RDN group. AMI indicates acute myocardial infarction; HF, high frequency; LF, low frequency; RDN, renal denervation.

Both low frequency (LF) norm and LF/high frequency (LF/HF) increased during AMI and 4 weeks after MI. The LF norm and LF/HF were significantly lower in the RDN group than in the sham group during the first 24 hours and at 4 weeks after MI (AMI, RDN versus sham: LF norm: 37.72±6.72 versus 51.09±8.96; *P*=0.0222; LF/HF: 0.97±0.29 versus 1.28±0.28; *P*=0.0227; LF norm at 4 weeks: 34.41±7.79 versus 45.48±9.80; *P*=0.0326; LF/HF at 4 weeks: 0.72±0.20 versus 1.05±0.30; *P*=0.0253; Figure 7A and 7C). However, the HF norm decreased slightly during AMI and 4 weeks after MI, with no significant differences between the RDN and sham groups (Figure 7B).

#### Nerve discharge

**Figure 8:**
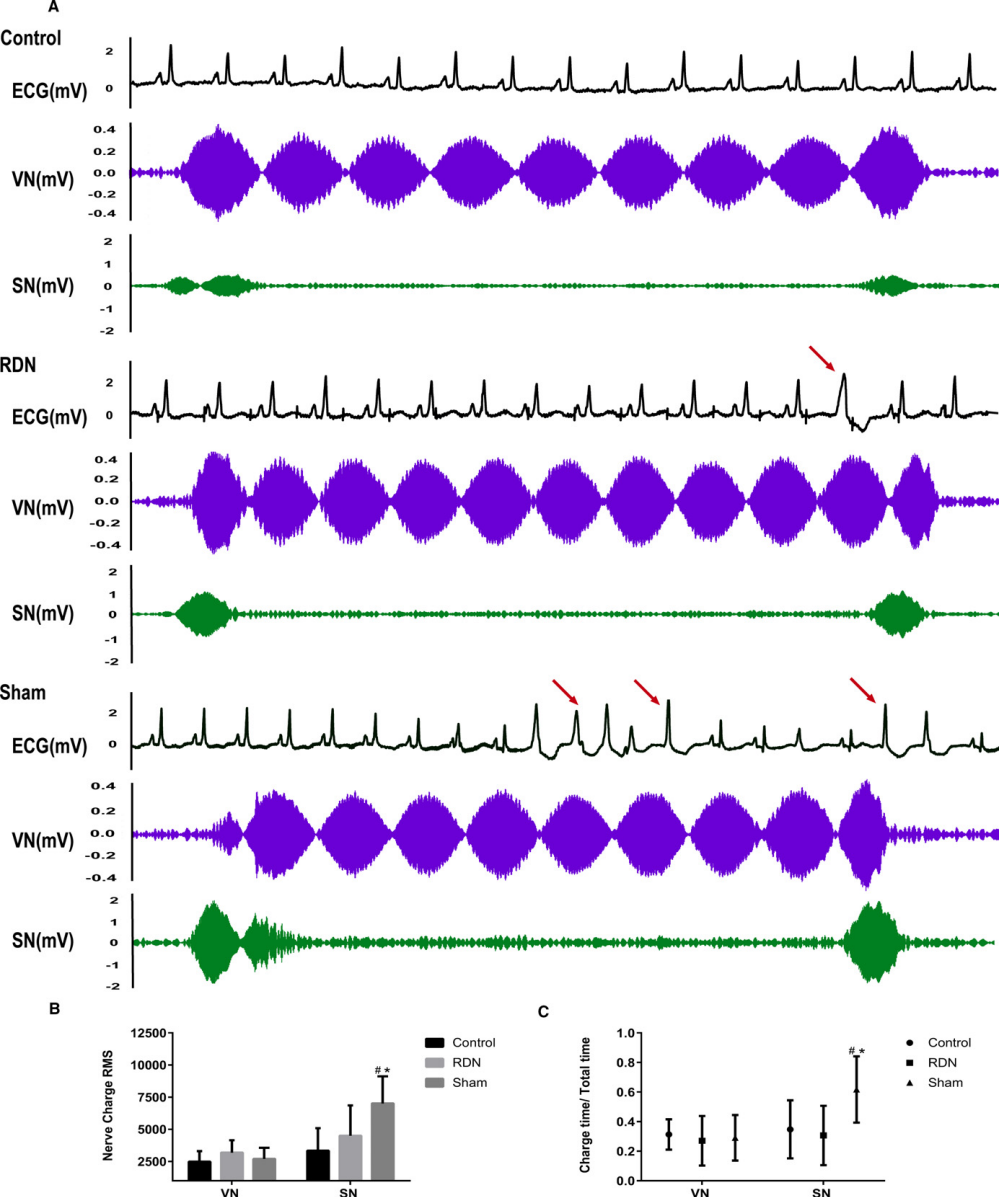
A, Representative ECG, VN discharge, and SN discharge in the control, RDN, and sham groups; ventricular arrhythmias are indicated with arrows. B and C, Nerve discharge measurement indexes: RMS and Dt/Tt in the control, RDN, and sham groups. ^#^ *P*<0.05 compared with the control group; *P<0.05 compared with the RDN group. Dt/Tt indicates proportion of nerve discharge duration of the total recording time course; RDN, renal denervation; RMS, root mean square; SN, sympathetic nerve; VN, vagus nerve.

Cardiac electrical activity, VN activity (cervical VN trunk), and postganglionic cardiac SN activity (emanating from the SG) were simultaneously recorded 4 weeks after MI. Representative ECGs and nerve discharge recordings for the 3 groups are presented in Figure 8A. No VA occurred in the control group, whereas several single VPBs were observed in the RDN group. In the sham group, spontaneous nonsustained VT and VPBs were recorded. There was no significant difference in VN discharge RMS among the 3 groups (control: 2476.36±832.27; RDN: 3191.28±953.63; sham: 2699.09±868.39; *P*=0.5969; Figure 8B). For SN discharge RMS, the control group displayed the minimum value, and the RDN group displayed a larger value than the control group, but the difference was not statistically significant. The RMS of the sham group was the largest, and the difference was marked relative to the RMS value of the other 2 groups (control: 3330.64±1753.6; RDN: 4503.188±2357.14; sham: 7009.24±2111.37; *P*=0.0195; Figure 8B). There was no difference in VN Dt/Tt among the 3 groups. Nevertheless, the SN Dt/Tt was evidently higher in the sham group than in both the control group and the RDN groups (median: control: 0.3474 [IQR: 0.1482–0.5638]; RDN: 0.3060 [IQR: 0.1367–0.5061]; sham: 0.6168 [IQR: 0.3927–0.8509]; *P*<0.0001; Figure 8C).

### Injury to the Renal Artery and Nerve After RDN

**Figure 9:**
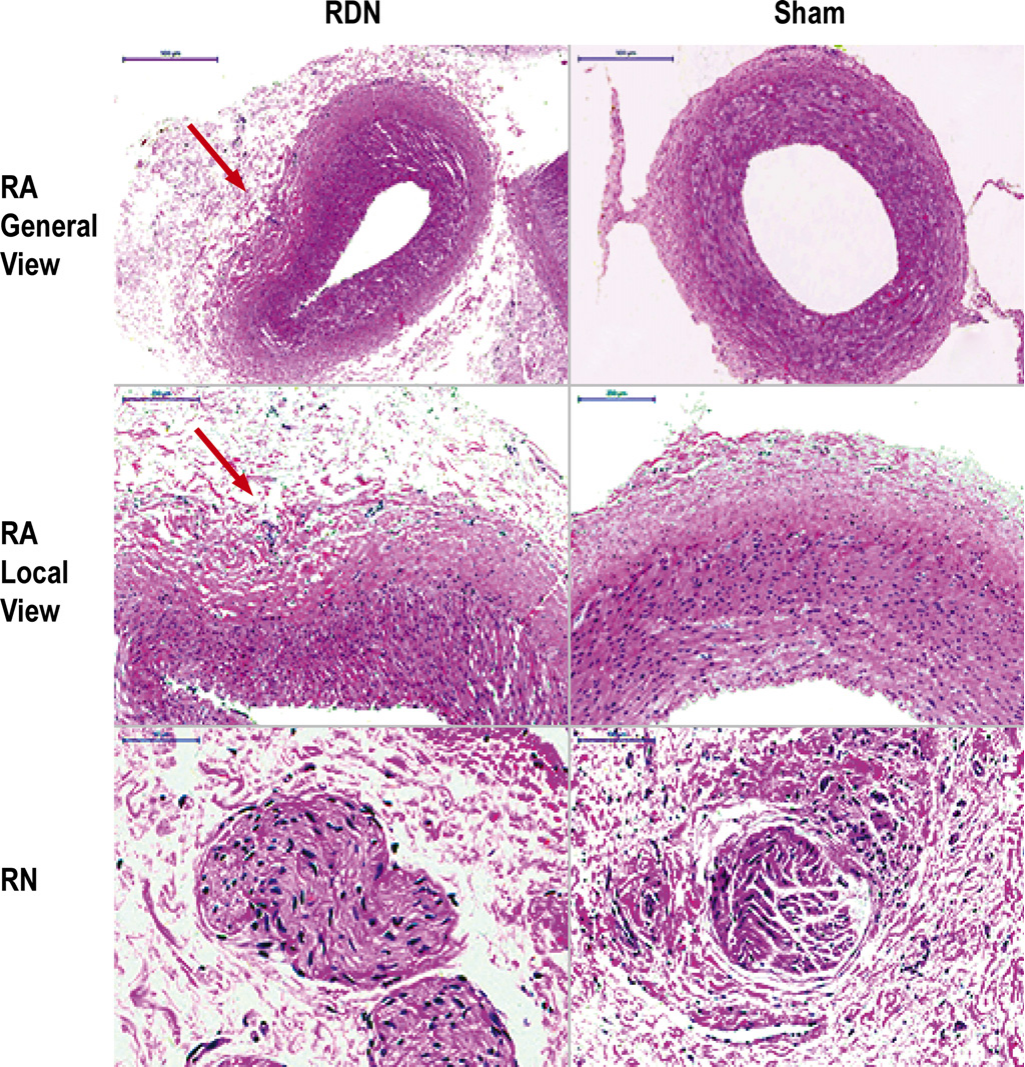
HE staining of RA in the RDN and sham groups (general and local view). RN fiber located at the renal adventitia of the RDN and sham groups. HE indicates hematoxylin and eosin; RA, renal artery; RDN, renal denervation; RN, renal nerve.

To assess injury to the renal artery and nerve, we observed the cross‐section of renal artery with hematoxylin and eosin staining. After RDN, the renal adventitia thickened and swelled, and the cells proliferated and became irregularly arranged. The renal nerve fiber located in the renal adventitia displayed morphological change, and obvious demyelination took place (Figure 9).

### Effect of RDN on Neural Remodeling

**Figure 10:**
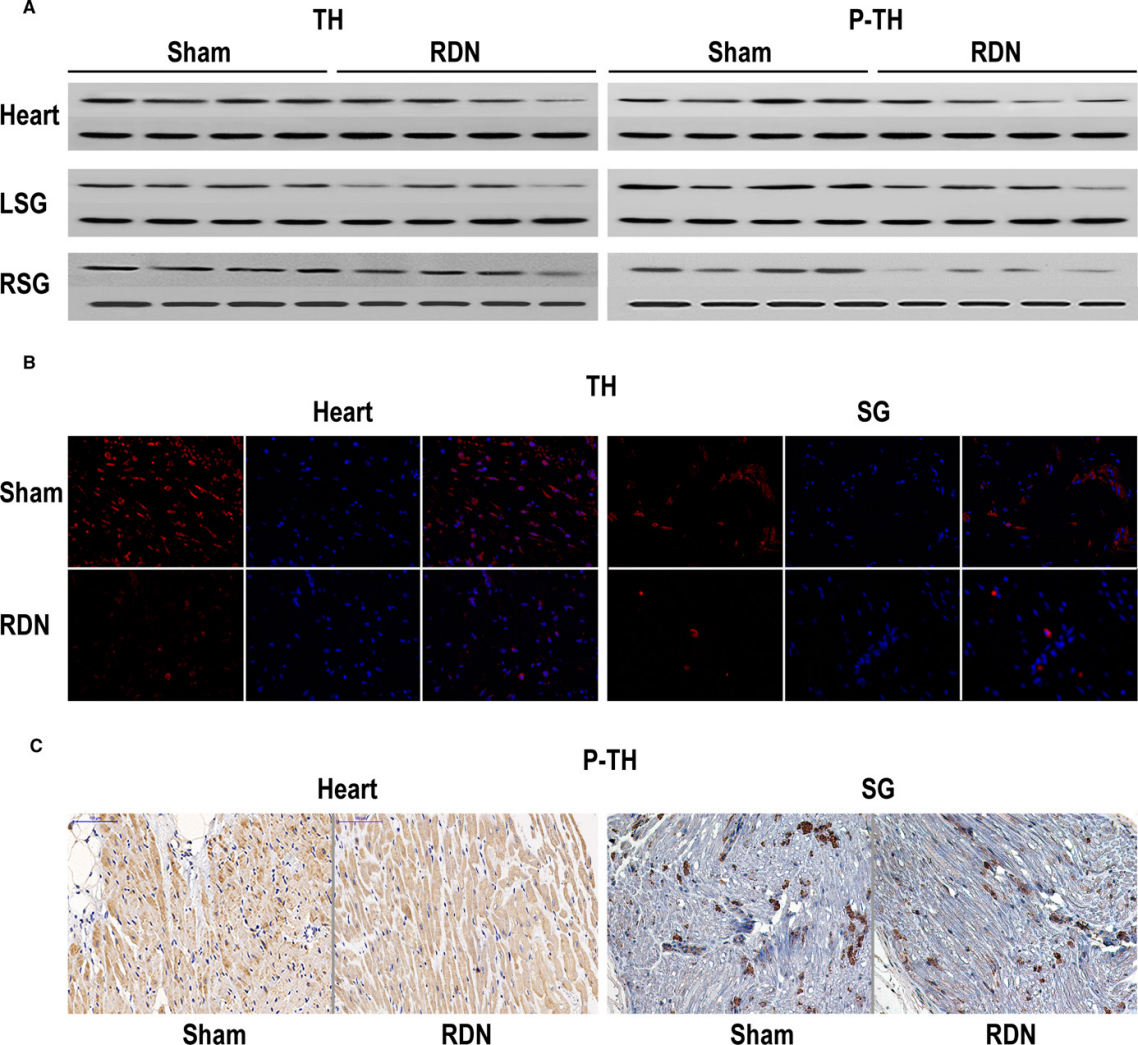
A, TH expression in the heart (IBZ) and bilateral SGs of the sham and RDN groups. B, TH‐positive nerves (immunofluorescence assay) in the heart (IBZ) and SG of the sham and RDN groups. C, P‐TH‐positive nerves (immunohistochemistry) in the heart (IBZ) and SG of the sham and RDN groups. GAPDH was used as the internal reference. IBZ indicates infarction border zone; P‐TH, phosphorylated form of tyrosine hydroxylase; RDN, renal denervation; SG, stellate ganglion; TH, tyrosine hydroxylase.

**Figure 11:**
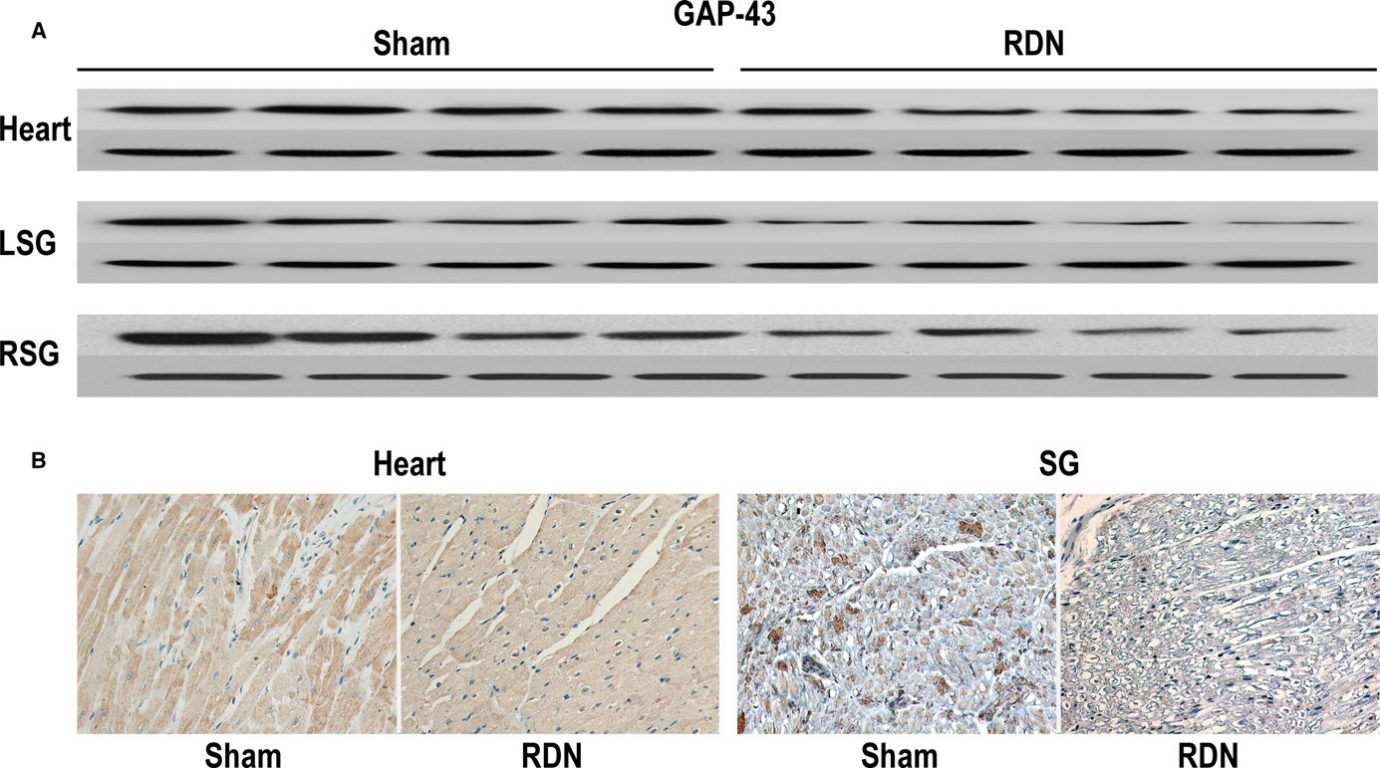
A, GAP‐43 expression in the heart (IBZ) and bilateral SGs of the sham and RDN groups. B, GAP‐43–positive nerves (immunohistochemistry) in the heart (IBZ) and SG of the sham and RDN groups. GAPDH was used as the internal reference. GAP‐43 indicates growth associated protein 43; IBZ, infarction border zone; LSG, left stellate ganglion; RDN, renal denervation; RSG, right stellate ganglion; SG, stellate ganglion.

As a specific marker of SN, tyrosine hydroxylase (TH) expression in the IBZ and bilateral SGs were analyzed by western blotting and immunofluorescence assay or immunohistochemistry. As shown in Figure 10A, TH protein expression was lower in the RDN group than in the sham group, not only in the heart (IBZ) but also in bilateral SGs. The phosphorylated form of TH was also lower in animals in the RDN group. Immunofluorescence assay showed lower levels of positive TH staining in the nerve of the IBZ and SGs in the RDN group than in the sham group, as shown in Figure 10B. The reduction in the distribution of phosphorylated TH‐positive nerves in the RDN group, as demonstrated by immunohistochemistry, is shown in Figure 10C. To assess remodeling of SN further, we compared the expression of GAP‐43 (growth associated protein 43), which is specific for growth cones of sprouting axons, between the RDN and sham groups. GAP‐43 expression was measured to identify growing nerves and was expected to be positive in sprouting nerves. In addition, the nerves that stain positive for GAP‐43 may not be mature enough to express TH; therefore, the combination of TH and GAP‐43 staining can offer the whole picture of the SN. GAP‐43 protein expression was decreased in the IBZ and SN (Figure 11A) of the RDN group. Immunohistochemistry of GAP‐43–positive nerves displayed differences between the RDN and sham groups and suggested reduced nerve sprouting in dogs in the RDN group. Quantification of the western blotting (Figure S2), immunofluorescence assay and immunohistochemistry staining results (Figure S3) was performed with Image‐Pro Plus (Media Cybernetics) and is presented in Data S1.

## Discussion

RDN was initially noted for its application in refractory hypertension. As an increasing number of researchers have realized that RDN plays its role by decreasing the whole‐body sympathetic tone, RDN has been widely adopted for treating many sympathetic‐driven diseases. RDN, for example, has been proven to be beneficial in heart failure,^20^ chronic kidney disease,^21^ pulmonary hypertension,^22^ insulin resistance,^23^ and arrhythmia.^24^

Although the clinical efficacy of RDN in refractory hypertension remains controversial, its performance in arrhythmia is promising. For atrial arrhythmia, in a prospective clinical study, 27 patients with atrial fibrillation and refractory hypertension who were referred for pulmonary vein isolation were randomized to pulmonary vein isolation only and pulmonary vein isolation combined with RDN. The results showed that RDN decreased the blood pressure and the recurrence of atrial fibrillation.^25^

For VA, RDN was first applied in 2 patients with chronic heart failure (one with nonobstructive hypertrophic cardiomyopathy and one with dilated cardiomyopathy) experiencing therapy‐resistant electrical storm; according to the follow‐up, ventricular tachyarrhythmia was significantly reduced in both patients.^26^ A similar study also demonstrated positive results in 4 patients with cardiomyopathy (2 nonischemic and 2 ischemic) with recurrent VT.^27^ A multicenter clinical study concluded recently that in patients with chronic heart failure, RDN was associated with reduced arrhythmic burden.^28^

In addition to the application of RDN for VA with underlying myopathy or heart failure, RDN has been reported to be used in patients with coronary heart disease with a history of MI. A 63‐year‐old male patient had acute ST‐segment–elevation MI. Despite successful percutaneous coronary intervention, the patient had repeated VT and VF refractory to drugs or ablation. However, the dramatically unmanageable VT and VF were ameliorated after RDN.^29^ Nevertheless, despite the marked efficacy of RDN, owing to the small sample size and the study design, these cases are limited in their stringency.

In this study, we established an animal model of MI. VA including VF was observed in both groups of animals within the first hour of MI. RDN evidently reduced VA, and this effect lasted up to 4 weeks after MI. More specifically, we demonstrated, first, that the renal artery and renal nerve were injured by surgical and chemical RDN, and this injury was sufficient to attenuate the blood pressure–increasing response induced by the electrical stimulation of ARG. Second, RDN reversed electrophysiological changes caused by MI, including shortened ERPs; increased ERP dispersion; and steepened the restitution curve, especially in the IBZ, thereby elevating the VF threshold and further reducing VAPMI. Third, as assessed by catecholamine contents of plasma and specific tissues, variability in the heart rate, and, more directly, recordings of discharge of the inferior cardiac SN, RDN could inhibit systemic SN activity and SN tones of the kidney and heart. Fourth, in addition, the suppressed SN activity was accompanied by decreased neural remodeling of the SG and IBZ, and this may have contributed to the efficacy of RDN in reducing VAPMI.

Epicardial electrophysiological studies have indicated that the ERP is considerably shortened after coronary ligation,^30^ although the decrease is not uniform throughout different locations. As described earlier, the most obvious change occurred in the IBZ. Even at 4 weeks after MI, the ERP at the IBZ was not restored as much as the ERPs at other locations. Nonsynchronous variations in ERP resulted large electrical variability across different locations and facilitated arrhythmia.^31^ The disintegration of reentrant wave fronts has been shown to result in VA. According to this restitution hypothesis, a key determinant of the wave‐front disintegration leading to VA, especially VF, is a steep electrical restitution curve in which small changes in the diastolic interval can produce large fluctuations in action potential duration and refractory period.^32^, ^33^ We also recorded MAPD and constructed a curve to explore the restitution characteristics. We concluded that RDN decreased ERP dispersion and flattened the restitution curve of MAPD, thereby modifying the electrophysiological basis for VAPMI.

MI has been shown to induce ventricular nerve injury^34^; and regeneration of the nerve occurs after damage.^35^ However, nerve regeneration can result in hyperinnervation of the IBZ^36^ and subsequent inhomogeneous nerve distribution, which might be the underlying mechanism for the inhomogeneous electrophysiological characteristics after MI. The study of native hearts of transplant recipients has demonstrated that regional reinnervation was associated with a history of spontaneous VA and might be responsible for the occurrence of VA and SCD in these patients.^6^ This causal relationship was further validated by an animal study in which nerve growth factor was infused to the left SG in dogs with chronic MI and an AV block was performed to augment SN sprouting and to create a successful model of spontaneous VT, VF, and SCD.^37^ Considering these results, SN sprouting may be an important determinant of VA and SCD in chronic MI.

In addition, in a canine model of MI, increased nerve activity associated with increased neuronal size and synaptic density in bilateral SGs was reported.^38^ A similar change was observed in a porcine model of MI.^39^ These results provided a possible mechanistic link between neural remodeling and VA.^40^ In our study, we assessed sympathetic neural remodeling by TH and GAP‐43 immunoreactivity and concluded that RDN reversed sympathetic neural remodeling in the IBZ and bilateral SGs. Consequently, for the treatment of VAPMI, the antineural remodeling effect of RDN may be an intervening pathway in addition to the inhibition of systemic sympathetic activity.

### Limitations

We aimed to observe the effect of RDN on spontaneous VAPMI, and the recording mode was weekly DEC. This intermittent recording may lead to the omission of some information. Implants that can simultaneously record multiple biological signals, including cardiac electrical activity, blood pressure, and neural discharge, would be an ideal way to obtain complete information. In addition, hematoxylin and eosin staining was used to observe injury to the renal nerve; however, it is not specific for nerves. Argentaffin staining may be a better choice for future studies.

## Sources of Funding

This study was supported by the Science and Technology Project of Xinjiang Uygur Autonomous Region (201517101).

## Disclosures

None.

## Supporting information


**Data S1.** Supplemental methods.
**Figure S1.** Blood pressure at baseline, after aorticorenal ganglia (ARG) stimulation, and after ARG stimulation following renal denervation.
**Figure S2.** Quantification of western blotting. Expression of tyrosine hydroxylase (TH), phosphorylated TH, and GAP‐43 (growth associated protein 43) in the heart (infarction border zone) and bilateral stellate ganglia of the sham and renal denervation groups was compared.
**Figure S3.** Quantification of tyrosine hydroxylase (TH)–positive nerves (immunofluorescence assay), phosphorylated TH–positive and GAP‐43 (growth associated protein 43)–positive nerves (immunohistochemistry) in the heart (infarction border zone), and stellate ganglion of the sham and renal denervation groups was compared.
