# Supplementary material for: Renal Denervation Reduced Ventricular Arrhythmia After Myocardial Infarction by Inhibiting Sympathetic Activity and Remodeling
Source: J Am Heart Assoc. 2018 Oct 13;7(20):e009938. doi: 10.1161/JAHA.118.009938 (PMC6474949; doi:10.1161/JAHA.118.009938)
Supplement: Supplementary file 1 — Data S1. Supplemental methods. Figure S1. Blood pressure at baseline, after aorticorenal ganglia (ARG) stimulation, and after ARG stimulation following renal denervation. Figure S2. Quantification of western blotting. Expression of tyrosine hydroxylase (TH), phosphorylated TH, and GAP‐43 (growth associated protein 43) in the heart (infarction border zone) and bilateral stellate ganglia of the sham and renal denervation groups was compared. Figure S3. Quantification of tyrosine hydroxylase (TH)–positive nerves (immunofluorescence assay), phosphorylated TH–positive and GAP‐43 (growth associated protein 43)–positive nerves (immunohistochemistry) in the heart (infarction border zone), and stellate ganglion of the sham and renal denervation groups was compared. [file JAH3-7-e009938-s001.pdf]

Supplemental Material

Data S1.

Supplemental Methods

Animal Studies

All animals (weight range: 12-15kg, age range: 1-3years) were anesthetized with sodium pentobarbital (20 mg/kg) and induced by ketamine (2mg/kg). After the animals were unresponsive, they were incubated and positive-pressure ventilated. Electrocardiograms, oxygen saturation and invasive blood pressure were continuously monitored throughout the study protocol. In RDN and sham group, left thoracotomy was performed and myocardial infarction was established by ligating the left anterior descending branch of coronary artery below the first diagonal branch.

Open-chest Electrophysiological Examination

Effective Refractory Periods (ERPs) were obtained using one decapolar catheter sutured on the epicardium along the left anterior descending branch. LEAD-7000 multi-channel electric physiological instrument system (Jinjiang electronic, China) were used to perform programmed stimulation. ERPs were measured by delivering an 8 consecutive stimuli (S1–S2=300 ms) and a premature stimuli (S1–S2) at 2 times the diastolic threshold. The coupling interval of the premature stimulus was decreased in 5 ms, and the ERP was defined as the longest coupling interval that fails to conduct. ERPs were obtained from five distributed sites. The difference between the longest ERPs and the shortest ERPSs at all 5 sites (infarction zone1, infarction zone 2, IBZ , noninfarction zone 1, noninfarction zone 2) was defined as ERPs dispersion.

Baseline electrophysiological parameters were determined during left ventricular epicardial pacing at a pacing cycle length (PCL) of 300ms at 2 times the diastolic threshold. To induce ventricular arrhythmia, the PCL was decremented in 10 ms steps until loss of 1:1 capture or induction of ventricular fibrillation (VF). Once VF was induced the animal was categorized as VF and the one not elicited was categorized as NVF.

Left ventricular epicardial pacing at PCL of 100ms with an increase of stimuli intensity steps of 1 V until VF was induced. Each stimulus lasted for 10-second and interrupted by a 30-second rest period before next test. VF threshold was defined as the minimum voltage required to produce sustained VF. Monophasic Action Potential (MAP) was acquired with one quadripolar sutured on the epicardium parallelly with the decapolar catheter mentioned above. And the MAP signals were recorded using the LEAD-7000 multi-channel electric physiological instrument system (Jinjiang electronic, China) and stored for offline analysis of restitution. During MAP recording pacing was performed. The pacing protocol consisted of 30 paced beats at each of the following cycle lengths in milliseconds: 400, 350, 300, 280, 260, 250, 240,230,220,210,200.

Duration of the MAP at 90% repolarization (MAPD90) was measured. Diastolic interval was also calculated, which was the time between the 90% repolarization time of the second-to-last beat to the activation time of the last beat. To obtain dynamic restitution curve, the MAPD90 of the last beat of each pacing cycle was plotted against the diastolic interval of the preceding beat. Sigmoidal curve fitting was used to capture the potentially biphasic shape of the cardiac restitution curve.

Nerve separation and discharge recording methods
Vertical paramecia incision located at the supraclavicular fossa was made to expose the stellate ganglion（SG）. Behind the subclavian artery and vertebral artery, and in the adipose tissue in front of the seventh cervical vertebra, a star-shaped SG was visible. The adipose tissue surrounding the SG was bluntly dissected with a glass dissecting needle to expose its branches and the inferior cardiac sympathetic nerve（ICSN）. A pair of modified bipolar neural recording electrodes were connected to the ICSN and cervical vagus trunk separately, and embedded in surrounding tissue to maintain the physiological state of the nerve. And the recording electrodes were connected to a PowerLab DP-301 Differential Amplifier (ML866/P; ADInstruments, Bella Vista, Australia) to record sympathetic nerve and vagus nerve discharges. Recorded signals from nerve discharges were band-pass filtered (high-pass 200 Hz, low-pass 120 Hz).

Four weeks later the last electrophysiological examination and nerve discharge recording were performed. Among the procedure, sodium pentobarbital (20 mg/kg) was continuously administrated through lateral vein of the forelimb. And at the time of sacrifice, the dose of sodium pentobarbital was added to 90mg/Kg.

Figure S1. Blood pressure at baseline, post-ARG stimulation, and post-ARG sitmulation after RDN.

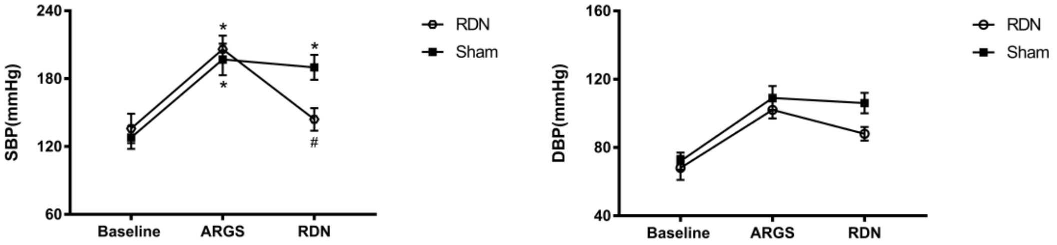

*P<0.05 compared to the baseline, #P<0.05 compared to ARG stimulation. ARG: aorticorenal ganglia; RDN:renal denervation.

Figure S2. Quantification of WB. Expression of TH, P-TH and GAP-43 in the heart (IBZ) and bilateral SGs of the sham and RDN groups was compared.

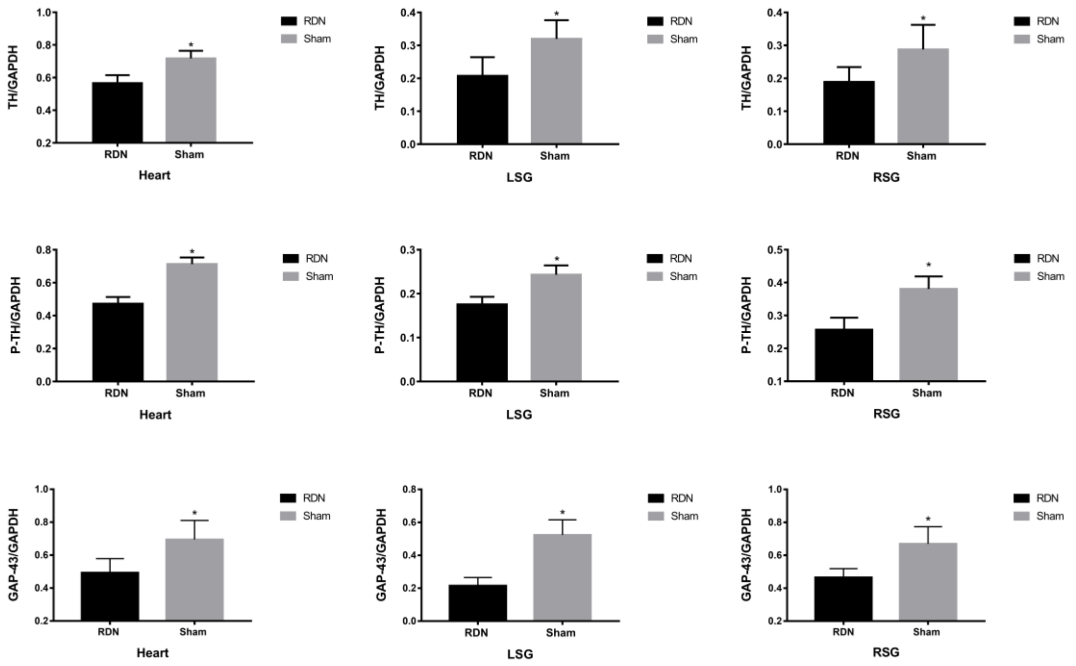

*P<0.05 compared to the RDN group. WB: TH: tyrosine hydroxylase; P-TH: phosphorylated form of tyrosine hydroxylase; GAP-43: growth associated protein-43; IBZ: infarction border zone; SG:stallate ganglion; RDN:renal denervation.

Figure S3. Quantification of TH-positive nerves (IFA), P-TH- and GAP-43-positive nerves (immunohistochemistry) in the heart (IBZ) and SG of the sham and RDN groups were compared.

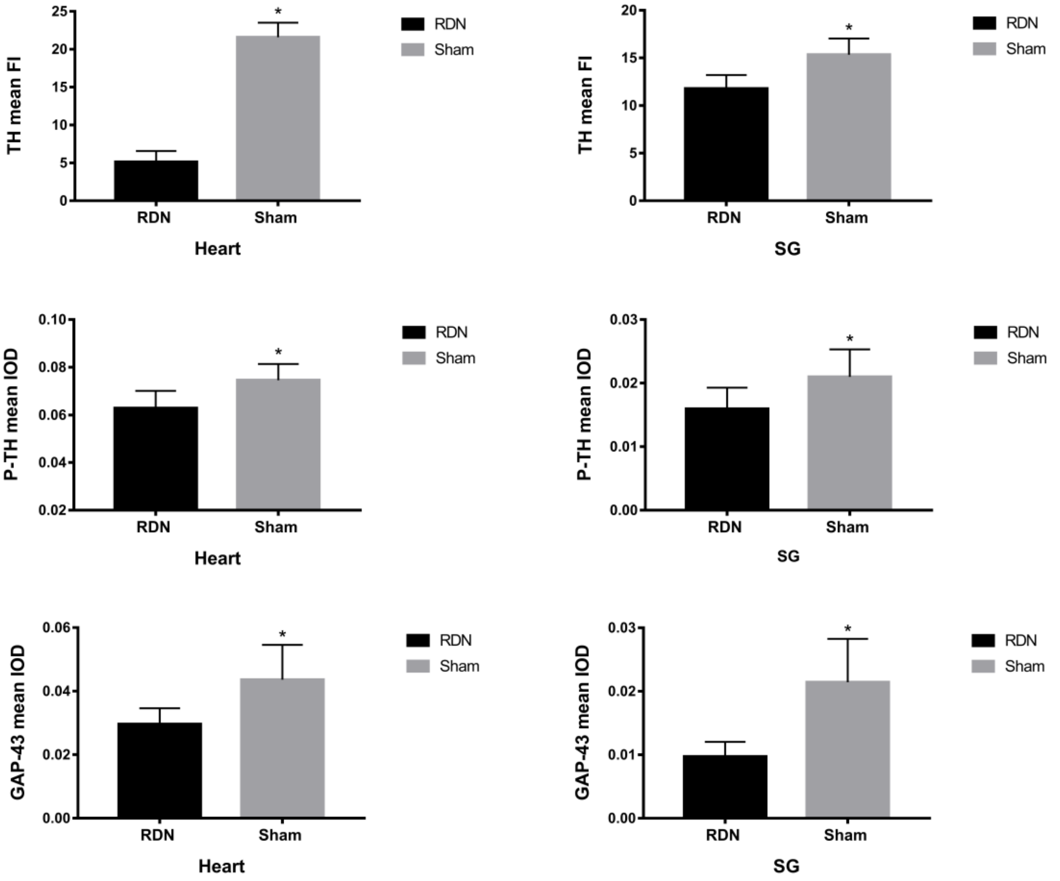

*P<0.05 compared to the RDN group. TH: tyrosine hydroxylase; IFA: immunofluorescence assay; P-TH: phosphorylated form of tyrosine hydroxylase; GAP-43: growth associated protein-43; IBZ: infarction border zone; SG:stallate ganglion; RDN:renal denervation; mean FI: mean fluorescence intensity; mean IOD: mean integrated optical density.
